# A population based study on human papillomavirus infection and associated risk factors among women of the remote South Andaman Island, India

**DOI:** 10.1186/s12905-024-02967-7

**Published:** 2024-02-23

**Authors:** Rehnuma Parvez, Paluru Vijayachari, Kannan Thiruvengadam, Avijit Roy, Mrinmoy Kumar Saha, Jawahar Ramasamy, Alwin Vins, Lipika Biswas, Alvencia Vaz, Harpreet Kaur, Muruganandam Nagarajan

**Affiliations:** 1 ICMR—Regional Medical Research Centre, Port Blair, 744103 India; 2 Directorate of Health Services, Port Blair, 744101 A&N Islands India; 3 G. B. Pant Hospital, ANIIMS, Port Blair, 744104 India; 4 Aarupadai Veedu Medical College and Hospital, Vinayaka Mission’s Research Foundation (DU), Pondicherry, 607402 India; 5 Department of Obstetrics and Gynecology, Arun Hospital, Port Blair, 744103 India; 6 Indian Council of Medical Research—Headquarters, New Delhi, 110029 India

**Keywords:** Human papilloma virus (HPV), Cervical cancer, Cervical cancer screening, South Andaman, Andaman and Nicobar Islands

## Abstract

**Background:**

Human papillomavirus (HPV) is associated with cervical cancer and cervical dysplasia worldwide. Data on HPV prevalence in a region is important because it serves as a predictor of the likelihood of the population in that particular region acquiring cervical cancer. Moreover, with the availability of effective vaccines, the public health system must be aware of the preponderance of HPV to implement the vaccine. The present study was designed to understand the prevalence of HPV and associated factors among the women of South Andaman Island.

**Methods:**

A cross-sectional study was conducted among married women of reproductive age (18–59 years) from South Andaman District from 2018 to 2022. Cervical scrapes were collected from participants after obtaining informed written consent for HPV molecular testing (HPV DNA) such as PCR assay. Demographic data was collected using a standard questionnaire and statistical analyses were performed to determine the associated factors.

**Results:**

The study showed prevalence of HPV as 5.9%(95% CI: 3.9–7.9) and prevalence of HR-HPV16 was 4.1% (95% CI 2.6 – 5.5) and HR-HPV18 prevalence was 1.8(95% CI: 0.6–3). The independent factors associated the HPV positivity were age above 55 years, menopause, post-menopausal bleeding, blood-stained vaginal discharge and loss of weight. Age was associated with all HPV infections among the South Andaman women.

**Conclusions:**

HPV 16 was reported as the predominant high risk HPV type circulating among women of South Andaman. Cervical cancer and precancerous lesions were significantly associated with HPV positivity and High risk HPV 16. Based on the knowledge of the risk factors associated with HPV, implementation of stronger public health awareness and prophylactic HPV vaccination is crucial among the women of this remote island.

## Introduction

One of the most prevalent malignancies in women worldwide is cervical cancer. In 2020, there were 604,000 new cases and overall 342,000 cases of cervical cancer. 90% of all new instances of cervical cancer occur in developing nations, placing a heavy burden on these nations according to World Health Organization (WHO) Factsheet, 2020 [1]. India reported a high incidence of cervical cancer, with incidence and death rates of 16.2% and 9.5%, respectively. As per WHO, the International Agency for Research on Cancer (IARC) report, 7.9 cases of cervical cancer per 100,000 people were reported in India. WHO was established an eradication programme to lower the incidence of cervical cancer to less than 4 instances per 100,000 females per year [2]. This cancer is most frequently concomitant to Human Papillomavirus (HPV). Even though the majority of pre-cancerous lesions resolve on their own, every woman is still at risk of HPV infection. High-risk human papillomavirus (HR-HPV) is linked with almost 99% of occurrences of cervical cancer as per the WHO factsheet, 2020 [3].

According to their risk of developing anogenital cancer, which includes cervical, vulvar, vaginal, and anal cancers, HPVs are classified into two types: high-risk (HR) types which are oncogenic and low-risk(LR) types which are non-oncogenic. There are approximately 200 distinct HPV genotypes known [4]. The LR-HPV types are typically linked to benign diseases like genital warts, but the HR-HPV types are linked to cancer [4, 5]. HPV 16, 18, 31, 33, 35, 39, 45, 51, 52, 56, 58, and 59 are high-risk types known to be linked to cervical cancer. HR- HPV 16, which is the most oncogenic type among all HR- HPVs is the main cause of cervical malignancies. 70% of cervical malignancies are related to HPV16 and HPV18 together [6]. About 90% of these lesions are caused by HPV16 and HPV18. Most frequently genital warts are linked to HPV6 and HPV11 [7].

According to Indian studies on cervical cancer, HPV positivity in invasive cervical carcinoma can reach 98%, with HPV type 16 accounting for more than 90% of these cases whereas high-grade squamous intraepithelial lesions (HSIL) had an HPV prevalence of 86.5% [8]. Most invasive cervical cancers were shown to have HPV16 as their primary cause (64.8%). HPV16 and HPV45 were more common in North India, whereas HPV35 seemed to be more common in South India [9]. The most common strain in Central India was HPV16. Other common HR- HPV types reported were HPV18, 31, 35, 45, 56, and 59, while HPV31, 51, 58, 59, 67, 82, and JEB2 were less common [10].

In addition to HPV infection, some cofactors like parity, early age of marriage, genital hygiene, sexual promiscuity, oral contraceptive use, smoking, immune-compromised status, other sexually transmitted infections and poor nutrition have been concomitant with cervical cancer [11].

Data on HPV prevalence in a region is important because it serves as a predictor of the likelihood of the population in that particular region acquiring cervical cancer. Moreover, with the availability of effective vaccines, the public health system must be aware of the preponderance of HPV to implement the vaccine [12].

The Andaman and Nicobar Islands are located nearer to Indonesia and Thailand in the southern parts of the “Bay of Bengal” in the Indian Ocean. The “territory” has a population of 380,581 people of whom 177,710 are women (46.7% of the total) as per the statistics in the Andaman and Nicobar administration. These islands are located far from mainland India [13]. The majority of the residents of these islands are from various parts of India, including six indigenous tribes with different socio-demographic and lifestyle characteristics [14].

In a previous study among a small subset of tribal and non-tribal women in these islands, HR- HPVs 16 and 18 were identified [14]. However, there is no comprehensive study on uterine cervical HPV prevalence among the women of Andaman and Nicobar Island. Hence the study aimed to estimate the prevalence of HPV and to understand the associated socio-demographic risk factors.

## Methodology

### Study design

This was a community-based cross-sectional study conducted from December 2018 to April 2022 among married women of age group (18 – 59 years) residing in the urban and rural areas of the South Andaman district of Andaman and Nicobar Islands, India. The reproductive age group of women is 15 to 49 years [15], but the persistence of HPV infection leading to cervical cancer is more in older age group [16], so women above 49 years age group were also included in the study.

Non-pregnant married women were included. Women who were pregnant, menstruating, postpartum, undergone hysterectomy /removal of cervix and unwilling to participate in the study were excluded from the study.

### Sample size

In this study, with the expected prevalence of human papillomavirus infection to be 60.3% (p) in an earlier study conducted by Senapati in 2017 [17]. With an absolute precision of 5% (d), and design effect of 2, the sample size (n) calculated by the using the below mentioned formula in OpenEpi version 3 [18].

$$\mathrm{Sample}\;\mathrm{size}\;\mathrm n\;=\;\left[\mathrm{DEFF}\ast\mathrm{Np}(1-\mathrm p)\right]/\left[(\mathrm d^2\star\mathrm Z_1^2-\alpha/2\ast(\mathrm N-1)+\mathrm p\ast(1-\mathrm p)\right]$$

The sample size was calculated as 736. Considering that the screening test is sensitive and invasive, we expected a high non-response rate and hence increased the sample size by 30% which comes to 957 and rounded off to 1000. As per the population census of the A & N Islands, the ratio between rural and urban areas is 2.5:1 in the population of Andaman [13]. Therefore the sample size was distributed between the rural and urban as 700 and 300 respectively. Then the Probability Proportional to Size (PPS) sampling was performed to choose the sampling unit cluster which is an village at the rural and ward at the urban strata.

### Data collection

The Field Clinic was set up at each cluster at the sub-centres, Primary Health Centres(PHCs), Community Health Centres (CHCs) and District Hospitals where all the participants were mobilised by the field investigators and evaluated using the pre-validated structured questionnaire closed ended questions which was divided into two sections. The first section captured potential information about the personal identity like name, address,contact details and socio-demographic determinants like education, occupation, monthly income. It also included questions seeking information on sexual behavior and reproductive characteristics which were assessed by asking questions on the sexual orientation, number of sexual partners, age of first sexual intercourse, also questions were asked about any history of Pelvic inflammatory disease (PID) and sexually transmitted diseases.The menstrual history, marital and obstetric history and menopausal details were also seeked for.

The second section of the questionnaire seeked for the gynaecological symtoms like bleeding per vagina, vaginal discharge, menorrhagia, dysmennorhoea,polymennorhoea, intermenstrual bleeding, chronic pelvic pain, post-menopausal bleeding, lower abdominal pain, painful sexual intercourse, painful micturion and loss of weight.

### Sample collection

Prior to requesting consent, participants were informed about the potential advantages and dangers of the research in local language. All participants who provided informed consent were instructed not to use any vaginal medication, lubricants, douches, vaginal contraceptives at least 48 h before the day of sample collection. Also to avoid sexual intercourse the night before sample collection. The cervical scrapes were collected using a cytobrush by a team of a clinician and trained Auxiliary Nurse and Midwife (ANM), placed in a conical vial with phosphate-buffered saline, and transported to the lab while maintaining a cold chain. Before putting the brush into PBS, the cytobrush containing the cervical scrapings was smeared on slides for Papanicolaou(Pap) test and slides were put in a Coplin jar with fixative for further cytopathological reporting by using the Bethesda System Reference [19].

### Laboratory tests

At the molecular laboratory of ICMR RMRC Port Blair samples were vortexed, centrifuged, and the nucleic acid was extracted using a QIAamp® DNA Mini Kit. Laboratory testing was conducted to validate DNA concentration using PCR for ß-globin as an internal control [11]. HPV DNA was identified using PCR with consensus primers, and HR-HPV16 and 18 were detected using previously published protocols [20].

### Ethical consideration

The study proposal was submitted and presented before the Human Ethics Committee on 29/06/2017 for seeking the permission before the commencement of the study. [IEC No: IEC/ICMR-RMRC/PB/Proj-03.Dated: 29/06/2017]. Following the approved guidelines of the Review Committee for Human Research, the study was executed.

### Statistical analysis

The profiles and clinical data were assessed using STATA 15.1 (StataCorp, Texas, USA). Data was shown using frequency and percentage. Bar charts were used to present the HPV symptoms. The crude prevalence and the 95% confidence interval were calculated based on the number of women screened in the survey and diagnosed with HPV infections. The adjusted prevalence was estimated using logistic regression, accounting for the cluster design, age-stratified participation rate in the survey, and population weights. The odds ratio was estimated and adjusted for the odds of reporting having HPV using a logistic regression model with related variables as explanatory variables. The explanatory variables such as socio-demographic, behavioral, sexual behavior, reproductive, and symptoms related to HPV positives were used for odds ratio estimation. Simple and multiple logistic regression models were used to estimate the odds ratio and adjusted odds ratio. The significance of all potential covariates was shown as an odds ratio with a 95% confidence interval. To identify the most influential variables, a multiple logistic regression model was performed using the stepwise method, with factors meeting the criteria of *p* ≥ 0.20 for removal from the model and *p* < 0.05 for addition to the model. The factors for the analysis were chosen based on the exploratory investigation by the simple logistic regression model and a literature review. All statistical analyses used two-tailed designs with a 0.05 significance level.

## Results

**Fig. 1:**
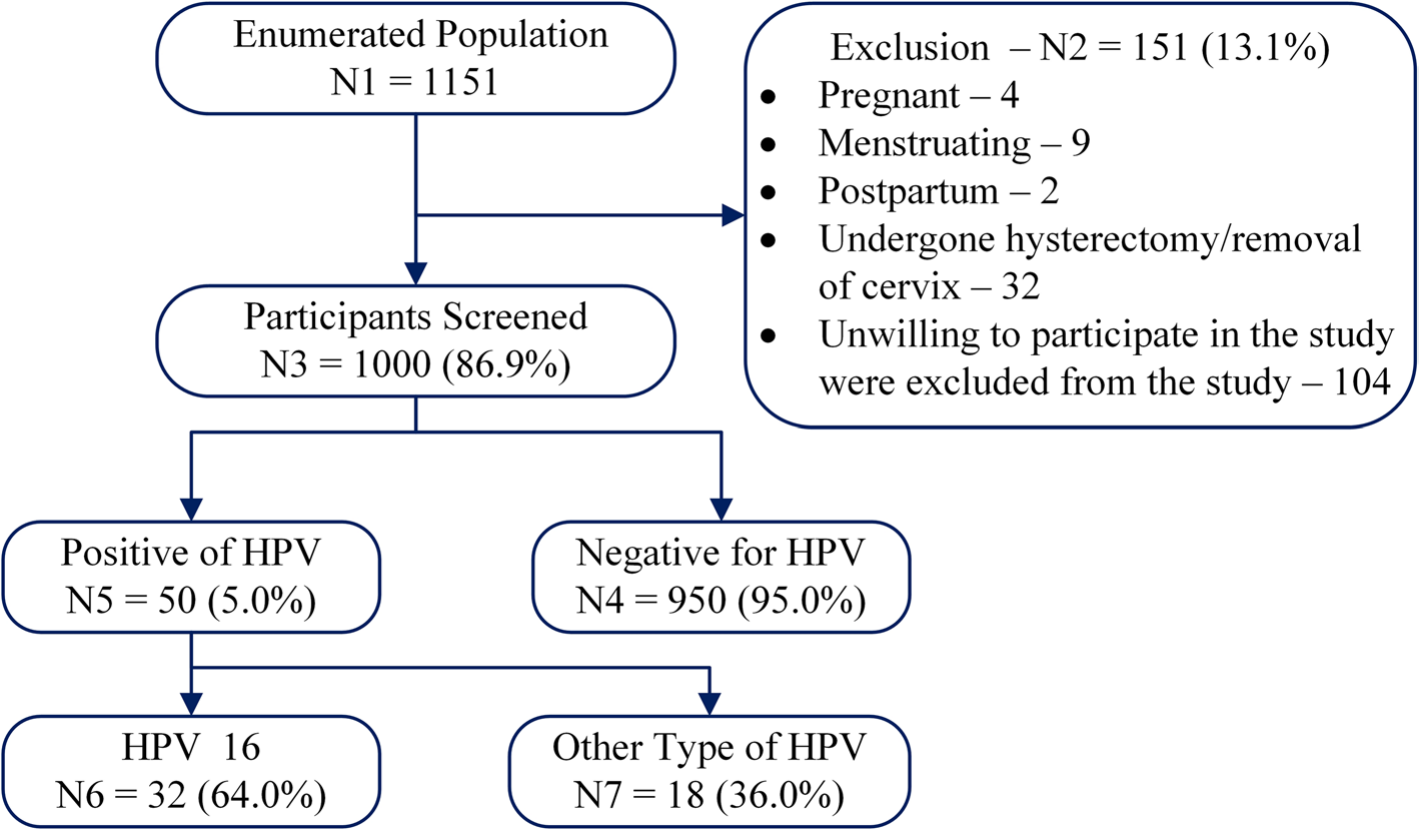
Flow chart of HPV infection status of survey participants

The study included 1151 women, of whom 151 (13.1%) declined to participate. The study enrolled 1000 (86.9%) women with an average age of 37.6 ± 9.2 years (Fig. 1). Among them, 72.7% resided in rural areas, with the majority having attained a high school (38.7%) or middle school (25.6%) education. 55.5% of participants were from the lowest socioeconomic class, and 25% reported tobacco use. Alcohol consumption was minimal (8.0%), and only 1.4% had a family history of cancer. Menarche occurred between 13–14 years for 71.3% of participants. Early marriage was common, with 44.3% marrying at 18–21 years and 17.8% before 18. 23.4% experienced their first pregnancy or conception before 20 years old. Most participants (54.8%) used family planning methods with tubal ligation being the most prevalent (32.5%). 15% reported condom use by male partners and 94.5% had only one sexual partner.

**Fig. 2:**
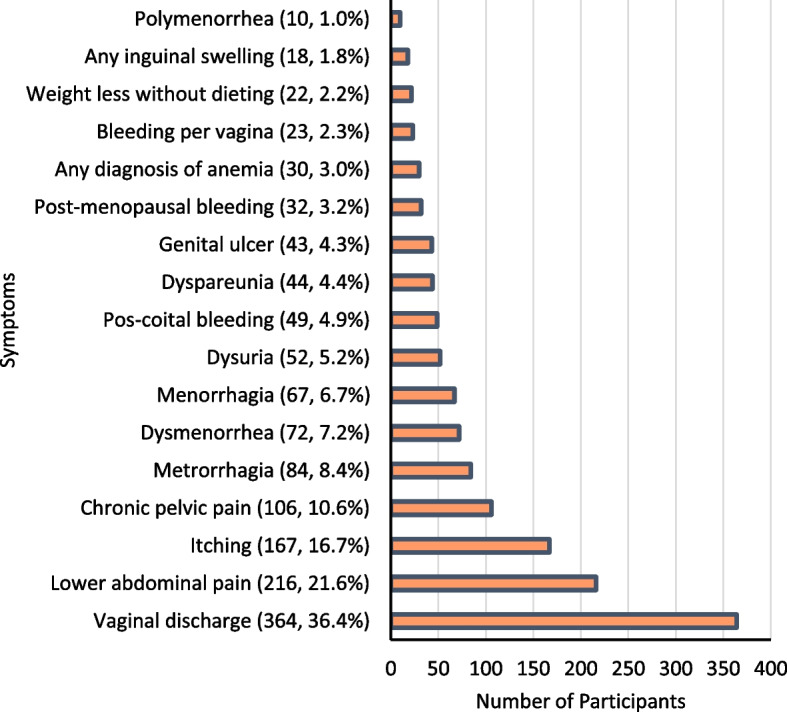
Beyond the virus: understanding the spectrum of HPV symptoms

Vaginal discharge was the most commonly reported symptom, affecting 364 women (36.4%). The majority reported white curdy discharge, while others experienced thin, watery, foul-smelling discharge. A smaller number reported blood-stained or greenish discharge. Lower abdominal pain ranked as the second most common symptom, affecting 216 women (21.6%). Chronic pelvic pain followed with 106 cases (10.6%). Other reported symptoms included intermenstrual bleeding, dysmenorrhea (painful periods), menorrhagia (heavy periods), dyspareunia (painful intercourse), dysuria (painful urination), post-coital bleeding, bleeding per vagina, and postmenopausal bleeding (Fig. 2).

The study revealed a 5.0% crude prevalence (95% CI: 3.7–6.5) of Human Papillomavirus (HPV) infection among 1000 women and adjusted prevalence of 5.9% (95% CI: 3.9–7.9). High-risk HPV (HR-HPV 16) was found in 3.2% (95% CI: 2.2–4.5) and adjusted prevalence was 4.1% (95% CI: 2.6–5.5) (Table 1). The age group 26–45 had the highest number of HPV-positive cases (35, representing 70% of the total), but this association was not statistically significant. On the other hand, women older than 55 were more likely to be HPV positive, but this association also did not reach statistical significance (aOR: 2.678, 95% CI: 0.583–12.302, *p* = 0.205). Similarly, residing in rural areas (33, 66% of HPV positives) and literacy status (40, 80% of HPV positives) showed no statistically significant association with HPV infection (OR: 1.353, *p* = 0.325 and OR: 1.189, *p* = 0.634, respectively). Interestingly, a majority of HPV-positive women were unemployed (41, 82%) and belonged to a lower socioeconomic class (27, 54%). However, these associations also lacked statistical significance. Notably, 35 women (70%) who were tobacco users had slightly higher odds of being HPV positive (OR: 1.51, *p* = 0.195), but this association was not statistically significant either (Table 2).

**Table 1:** Prevalence of HPV Infection, HPV-16 and other type of HPV

	**HPV Infection**	**HPV 16**	**Other HPV**
Screened	1000	1000	1000
Positive	50 (5.0%)	32 (3.2%)	18 (1.8%)
Crude prevalence per 1000 (95% CI)	50 (37, 65)	32 (22, 45)	18 (11, 28)
Adjusted prevalence per 1000 (95% CI)	59 (39, 79)	41 (26, 55)	18 (6, 30)

The adjusted prevalence was estimated after adjusting for the clustering effects, and age stratified participation rate and population weight

**Table 2:** The association of participants characteristics with human papillomaviruses

	**Negative**		**Positive**		**OR [95% CI]**	***p*** **-Value**	**aOR [95% CI]**	***p*** **-Value**
	**n**	**%**	**n**	**%**				
**Age Classification**								
18–25	82	8.6	4	8.0	Reference		Reference	
26–35	338	35.6	19	38.0	1.15 [0.38, 3.48]	0.801	1.05 [0.34, 3.22]	0.930
36–45	355	37.4	16	32.0	0.92 [0.30, 2.84]	0.890	0.81 [0.25, 2.55]	0.723
46–55	144	15.2	6	12.0	0.85 [0.23, 3.12]	0.811	0.73 [0.19, 2.83]	0.657
> 55	31	3.3	5	10.0	3.30 [0.83, 13.12]	0.089	2.67 [0.58, 12.3]	0.205
**Residential Area**								
Rural	688	72.4	33	66.0	Reference		Reference	
Urban	262	27.6	17	34.0	1.35 [0.74, 2.47]	0.325	1.33 [0.67, 2.63]	0.405
**Literate Status**								
Literate	785	82.6	40	80.0	Reference		Reference	
Illiterate	165	17.4	10	20.0	1.18 [0.58, 2.43]	0.634	1.07 [0.47, 2.43]	0.858
**Employment Status**								
Employed	179	18.8	9	18.0	Reference		Reference	
Unemployed	771	81.2	41	82.0	1.05 [0.51, 2.22]	0.882	1.15 [0.47, 2.78]	0.755
**Socio-economic Status Classification**								
Lower [0–10]	528	55.6	27	54.0	Reference		Reference	
Middle [11–15]	292	30.7	16	32.0	1.07 [0.57, 2.02]	0.831	1.18 [0.59, 2.35]	0.628
Upper [> 15]	130	13.7	7	14.0	1.05 [0.45, 2.47]	0.906	1.36 [0.47, 3.89]	0.562
**Do you use any form of tobacco (smoking/ smokeless)?**								
No	740	77.9	35	70.0	Reference		Reference	
Yes	210	22.1	15	30.0	1.51 [0.81, 2.82]	0.195	1.50 [0.76, 2.96]	0.240
**Do you consume alcoholic beverages?**								
No	943	99.3	49	98.0	Reference		Reference	
Yes	7	0.7	1	2.0	2.74 [0.33, 22.79]	0.349	2.22 [0.24, 20.37]	0.478
**Do you engage in any form of physical activity?**								
No	899	94.6	45	90.0	Reference		Reference	
Yes	51	5.4	5	10.0	1.95 [0.75, 5.15]	0.173	1.72 [0.60, 4.88]	0.304
**Do you have history of any illness?**								
No	733	77.2	37	74.0	Reference		Reference	
Yes	217	22.8	13	26.0	1.18 [0.62, 2.27]	0.605	0.90 [0.41, 1.99]	0.810
**Having a Diabetes**								
Non-Diabetic	849	89.4	44	88.0	Reference		Reference	
Diabetic	101	10.6	6	12.0	1.14 [0.48, 2.76]	0.761	1.13 [0.41, 3.11]	0.811
**Having a Hypertension**								
Non-Hypertension	742	78.1	38	76.0	Reference		Reference	
Hypertension	208	21.9	12	24.0	1.12 [0.58, 2.20]	0.726	1.28 [0.63, 2.62]	0.482
**Having a Tuberculosis**								
Non-Tuberculosis	947	99.7	49	98.0	Reference		Reference	
Tuberculosis	3	0.3	1	2.0	6.44 [0.66, 63.07]	0.110	6.24 [0.50, 76.54]	0.152

*OR* Odds Ratio, *aOR* Adjusted Odds Ratio

Among the positives, 32 (64%) had their sexual debut before age 20, but this association was not statistically significant. 48 (96%) had only one sexual partner, indicating a low association with HPV positivity. 37 (74%) reported regular menstrual cycles, suggesting no significant connection to HPV infection. Interestingly, having living children appeared protective against HPV. Among HPV-positive women, those with children (39, 78%) had lower odds of being HPV-positive compared to those without children (OR: 0.418, *p* = 0.017). Another potentially protective factor emerged in menstrual pad usage. HPV-positive women who used less than four pads per day (39, 78%) had higher odds of HPV compared to those using more (OR: 0.235, *p* = 0.036). This association was statistically significant. Women who were postmenopausal had higher odds of being HPV-positive (aOR: 2.17, *p* = 0.047), showing a statistically significant association. Early marriage (at 21 years or less) was associated with higher HPV odds (OR: 0.73, *p* = 0.371) compared to marrying after 21 (OR: 0.34, *p* = 0.032). This association was also statistically significant (Table 3).

**Table 3:** The association of reproductive characteristics of the participants with human papillomaviruses

	**Negative**		**Positive**		**OR [95% CI]**	***p*** **-value**	**aOR [95% CI]**	***p*** **-value**
	**n**	**%**	**n**	**%**				
**Age at first intercourse**								
< 16 Years	51	5.4	4	8.0	Reference		Reference	
16–20 Years	461	48.5	28	56.0	0.77 [0.26, 2.30]	0.645	0.88 [0.28, 2.75]	0.829
20–45 Years	438	46.1	18	36.0	0.52 [0.17, 1.61]	0.259	0.67 [0.18, 2.42]	0.543
**No of Sexual Partners**								
One Partner	897	94.4	48	96.0	Reference		Reference	
> 1 Partners	53	5.6	2	4.0	0.71 [0.17, 2.98]	0.635	0.88 [0.20, 3.80]	0.865
**Menstrual Cycle**								
Regular	652	68.6	37	74.0	Reference		Reference	
Irregular	298	31.4	13	26.0	0.77 [0.40, 1.47]	0.425	0.61 [0.31, 1.22]	0.168
**How many sanitary pads used in a day?**								
Nil	97	10.2	8	16.0	Reference			
1–2 Pads	177	18.6	10	20.0	0.69 [0.26, 1.79]	0.441		
3–4 Pads	521	54.8	29	58.0	0.68 [0.30, 1.52]	0.343		
> 4 Pads	155	16.3	3	6.0	0.24 [0.06, 0.91]	0.036		
**Have you attained menopause?**								
No	831	87.5	40	80.0	Reference		Reference	
Yes	119	12.5	10	20.0	1.75 [0.85, 3.58]	0.129	2.16 [1.01, 4.65]	0.047
**Age at Marriage**								
< 18 years	165	17.4	13	26.0	Reference			
18 – 21 years	419	44.1	24	48.0	0.73 [0.36, 1.46]	0.371		
22 – 25 years	225	23.7	6	12.0	0.34 [0.13, 0.91]	0.032		
26 – 29 years	91	9.6	4	8.0	0.56 [0.18, 1.76]	0.320		
> 30 years	50	5.3	3	6.0	0.76 [0.21, 2.78]	0.680		
**Age at first Pregnancy**								
Never Pregnant	77	8.1	5	10.0	Reference		Reference	
< 20 Years of Age	217	22.8	17	34.0	1.21 [0.43, 3.38]	0.721	1.27 [0.39, 4.05]	0.685
≥ 20 Years of Age	656	69.1	28	56.0	0.66 [0.25, 1.75]	0.402	0.73 [0.26, 2.01]	0.544
**Number of Conceptions**								
Zero	85	8.9	5	10.0	Reference			
1 to 2	619	65.2	33	66.0	0.91 [0.34, 2.39]	0.842		
≥ 3	246	25.9	12	24.0	0.83 [0.28, 2.42]	0.732		
**Number of Living Children**								
Zero	112	11.8	11	22.0	Reference			
1 to 2	731	76.9	30	60.0	0.42 [0.20, 0.86]	0.017		
≥ 3	107	11.3	9	18.0	0.86 [0.34, 2.15]	0.741		
**Are you following any family planning methods?**								
No	424	44.6	28	56.0	Reference		Reference	
Yes	526	55.4	22	44.0	0.63 [0.36, 1.12]	0.118	0.55 [0.29, 1.01]	0.055

*OR* Odds Ratio, *aOR* Adjusted Odds Ratio

Among those with HPV, 32% (16) reported vaginal discharge, but this symptom was not statistically linked to HPV positivity. However, 12% (6) of women with HPV experienced postmenopausal bleeding, and they were about six times more likely to be HPV-positive compared to those without it (aOR: 5.85, *p* = 0.014). This significant association suggests postmenopausal bleeding as a potential indicator for HPV infection in this age group. A smaller group of women (8%, 4) had blood-stained vaginal discharge, which was associated with a 5.44 times higher chance of being HPV-positive compared to those without it (OR: 5.45, *p* = 0.011). This significant association further strengthens the potential link between this symptom and HPV infection. Although other symptoms were not statistically significant, they showed trends toward increased HPV risk, such as chronic pelvic pain, lower abdominal pain, itching, dyspareunia, and painful urination. Notably, participants experiencing weight loss without dieting had 3.82 times higher odds of being HPV-positive compared to those with no weight loss (aOR: 3.862, *p* = 0.047). This statistically significant association suggests weight loss as a potential indicator or risk factor for HPV infection (Table 4).

**Table 4:** The association of symptomatic characteristics of the participants with human papillomaviruses

	**Negative**		**Positive**		**OR [95% CI]**	***p*** **-Value**	**aOR [95% CI]**	***p*** **-Value**
	**n**	**%**	**n**	**%**				
**Any post-menopausal bleeding?**								
No	602	63.4	34	68.0	Reference		Reference	
Yes	348	36.6	16	32.0	4.84 [1.89,12.37]	0.001	5.85 [2.17, 15.76]	< 0.001
**Any complaints of Vaginal discharge?**								
No	939	98.8	47	94.0	Reference		Reference	
Yes	11	1.2	3	6.0	0.81 [0.44,1.49]	0.508	0.75 [0.31, 1.78]	0.522
**Blood Stained in the Vaginal Discharge**								
Nil	765	80.5	44	88.0	Reference		Reference	
Blood Stained	185	19.5	6	12.0	5.44 [1.47,20.19]	0.011	6.44 [1.31, 31.61]	0.022
**White Curdy in the Vaginal Discharge**								
Nil	886	93.3	47	94.0	Reference		Reference	
White Curdy	64	6.7	3	6.0	0.56 [0.23,1.34]	0.196	0.58 [0.18, 1.82]	0.353
**Any complaints of Menorrahagia?**								
No	882	92.8	46	92.0	Reference		Reference	
Yes	68	7.2	4	8.0	0.88 [0.26,2.91]	0.839	0.94 [0.27, 3.22]	0.932
**Any complaints of intermenstrual bleeding (metrorrhagia)?**								
No	869	91.5	47	94.0	Reference		Reference	
Yes	81	8.5	3	6.0	0.68 [0.20,2.24]	0.533	0.53 [0.14, 1.91]	0.333
**Any complaints of Chronic Pelvic Pain?**								
No	852	89.7	42	84.0	Reference		Reference	
Yes	98	10.3	8	16.0	1.65 [0.75,3.62]	0.208	1.5 [0.62, 3.59]	0.358
**Any complaints of Post-menopausal Bleeding?**								
No	930	97.9	49	98.0	Reference		Reference	
Yes	20	2.1	1	2.0	0.94 [0.12,7.21]	0.960	0.72 [0.08, 6.34]	0.769
**Any Complaints of Lower Abdominal Pain?**								
No	749	78.8	35	70.0	Reference		Reference	
Yes	201	21.2	15	30.0	1.59 [0.85,2.98]	0.142	1.23 [0.59, 2.54]	0.570
**Presence of Any Genital Ulcer?**								
No	910	95.8	47	94.0	Reference		Reference	
Yes	40	4.2	3	6.0	1.45 [0.43,4.86]	0.546	2.1 [0.55, 7.96]	0.272
**Any complaints of Itching?**								
No	796	83.8	37	74.0	Reference		Reference	
Yes	154	16.2	13	26.0	1.81 [0.94,3.49]	0.074	1.74 [0.83, 3.66]	0.139
**Any Complaints of Dyspareunia?**								
No	909	95.7	47	94.0	Reference		Reference	
Yes	41	4.3	3	6.0	1.41 [0.42,4.73]	0.573	1.41 [0.38, 5.22]	0.604
**Any complaints of Post Coital Bleeding?**								
No	902	94.9	49	98.0	Reference		Reference	
Yes	48	5.1	1	2.0	0.38 [0.05,2.83]	0.348	0.21 [0.02, 1.82]	0.160
**Have you lost weight without Dieting?**								
No	931	98.0	47	94.0	Reference		Reference	
Yes	19	2.0	3	6.0	3.12 [0.89,10.94]	0.074	3.63 [0.95, 13.78]	0.058
**Any Complaints of Painful Micturation?**								
No	901	94.8	47	94.0	Reference		Reference	
Yes	49	5.2	3	6.0	1.17 [0.35,3.90]	0.794	0.89 [0.24, 3.24]	0.866

*OR* Odds Ratio, *aOR* Adjusted Odds Ratio

A multivariable analysis revealed several factors significantly associated with HPV infection. Participants aged 55 and older were almost three times more likely to be HPV-positive compared to younger women (aOR: 3.23, 95% CI: 1.20–8.73, *p* = 0.021). Additionally, women who had reached menopause had twice the odds of HPV infection compared to those who hadn't (aOR: 2.232, 95% CI: 1.043–4.776, *p* = 0.039). Notably, those who had their first pregnancy at or after age 20 had lower odds of HPV infection (aOR: 0.55, 95% CI: 0.31–0.76, *p* = 0.041), indicating a potential protective effect. Postmenopausal bleeding was strongly associated with HPV infection, as women experiencing this were nearly three times more likely to be HPV-positive (aOR: 2.99, 95% CI: 1.35–6.63, *p* = 0.007). Furthermore, blood-stained vaginal discharge emerged as a significant risk factor, with women exhibiting this symptom being about five times more likely to be HPV-positive (aOR: 5.41, 95% CI: 1.34–21.88, *p* = 0.018). Finally, participants reporting weight loss without dieting had a fourfold increased risk of being HPV-positive, suggesting a potential link between these factors (Table 5).

**Table 5:** Multivariable model for risk factors includes the socio-demographic, behavioural, sexual behaviour, reproductive and symptoms associated with HPV positivity

Factor	aOR [95% CI]	*p*-Value
Age > 55	3.23 [1.20, 8.73]	0.021
Physical Activity	1.90 [0.72, 5.03]	0.194
Irregular Menstrual Cycle	0.62 [0.31, 1.22]	0.164
Attained Menopause	2.23 [1.04, 4.78]	0.039
First Pregnancy Age ≥ 20	0.55 [0.31, 0.98]	0.041
Adopting Family Planning Methods	0.60 [0.33, 1.07]	0.083
Post-Menopausal Bleeding	5.38 [2.06, 14.01]	0.007
Blood Stained in the Vaginal Discharge	6.32 [1.56, 25.55]	0.018
White Curdy in the Vaginal Discharge	0.50 [0.20, 1.23]	0.192
Post Coital Bleeding	0.24 [0.03, 1.81]	0.143
Itching Complaint	1.78 [0.89, 3.59]	0.112
Loss of Weight	3.86 [1.05, 14.21]	0.021

*p*-Value < 0.05—statistically significant

*aOR* Adjusted Odds Ratio

Among the HPV positives, eight were reported to have epithelial abnormalities in the Pap smear. HR-HPV16 was found to be significantly associated with abnormalities like Low- grade squamous intraepithelial lesion LSIL, High- grade squamous intraepithelial lesion HSIL and Squamous cell carcinoma SCC reported in the Pap smear (*P*-value = 0.049). Also, HPV positivity was found to be significantly associated with these cytological cell abnormalities in Pap smears (*p* = 0.015).

In our study, among the 50 HPV positives, four (04) cases of cervical cancer were identified. All these cases were referred to the gynaecologist for the further treatment. One among these 04 cases was detected with cervical cancer Stage 4B and referred to mainland India for treatment but unfortunately patient expired. Out the rest of cases, one case who was detected with Stage 2B got referred to at Chennai and was completely treated. 02 cases diagnosed with Stage 3A were also referred and are under treatment presently. All the HPV positives identified were referred for further treatment to G.B. Pant Hospital which is the only tertiary care hospital in this island. Moreover the list of HPV positives were also communicated confidentially to Directorate of Health Services, Andaman and Nicobar Islands for follow up.

## Discussion

This was a cross-sectional community-based study reporting the HPV infection in the uterine cervix of women in the South Andaman district of Andaman and Nicobar Islands. Cervical morphology is altered by human papillomavirus infection, progressing from healthy cervical cytology to precancerous growths and ultimately invasive cervical carcinoma [21]. Evidence suggests that HPV testing is a useful method for detecting cervical malignancies, especially in population-driven cervical cancer surveillance programmes. Hence the information on the prevalence of HPV infection is extremely significant in a geographic area as it is a predictive tool for the likelihood of participants in that area developing cervical cancer [12]. This epidemiological data is crucial for implementing the appropriate preventive measures, particularly vaccination [22]. Vaccination effectiveness is also need to be assessed among various region [22]. A significant correlation of TNFA rs361525 polymorphisms with oral pre-cancer in the North Indian population and with reproductive tract infections in women has been reported [23].

The prevalence of HPV among asymptomatic women having normal cervical cytology was reported to be an average of 9.4% in Asia whereas a higher prevalence of 30.9% was reported in Oceania. HPV prevalence rate of more than 20% was reported in Africa North America, and South and Central America [24–26]. Studies from India suggested HPV prevalence ranging from 2.3% and 36.9%. There are few community-based studies from India screening sexually active women who appear to be normal using PCR-based methods. PCR methods performance also varies and its depends on samply types and sets of primers including other factors [27]. Some studies found that the frequency of HPV varied greatly throughout India, from 4.7% in Kolkata to 6.1% in the south to 19.2% among indigenous women in central India [28–30]. Our study demonstrated an HPV infection prevalence rate of 5.9% (95% CI 3.6 –6.4) which is similar to the prevalence of HPV, documented by community-based studies in India [12].

The present study reported HR- HPV16 as the most prevalent genotype in South Andaman. Similarly, HPV16 was the most prevalent genotype reported in Central India, Madhya Pradesh, Tamil Nadu and Andhra Pradesh [31–35]. An estimated 4.1% of the population had HR-HPV16 association which is comparable to the 3.8% prevalence of HPV 16 in a study conducted in a rural district of Tamil Nadu [29]. HPV18 had the second-highest prevalence, in our study which was reported in 4 (0.4%) cases. HPV16 & HPV18 together accounted for 36 (56%) cases among the total HPV genotypes circulating. Other research has also shown HPV 18 to be the second most often found high-risk HPV strain [35].

Previous research has recorded that once sexual activity commences, the probability of HPV infection elevates [36, 37]. In our study also the HPV positives were highest in the age group 26–45 years. A similar higher infection rate in this age group was reported in Odisha [17]. Though the number of HPV positives was highest among 26–45 years, the study participants more than 55 years of age had almost thrice the odds of developing HPV infection which was significantly associated with *p*-value < 0.05. Earlier studies had also reported that an age greater than 50 is significantly associated with HPV infection. The probable reason for this accociation could be the decrease in immune responses caused by hormonal changes which suppress the immunomodulation of the virus leading to HPV persistence or sometimes reactivation of HPV latent infection [23, 38, 39].

The symptoms like post-menopausal bleeding and HPV infection were both shown to be strongly correlated with each other. Considering the vulnerability of the postmenopausal women HPV, screening for HPV and cervical cancer should not exclude the elderly women and they should continue to be tested for HPV. In the current research, it was also shown that additional symptoms, including weight loss and vaginal discharge that was blood-stained, were considerably related to HPV infection.

Young women are more likely to contract the human papillomavirus (HPV), as an immature cervix provides favourable conditions for HPV [40, 41]. Our study also reported more chances of HPV infection among women who had early marriages at the age of 21 years or below compared to those who got married after 21 years of age.

Poor genital hygiene and prolonged use of sanitary pads have been shown to increase bacterial infections and yeast infections which significantly increases the risk of HPV infections [42–44]. Likewise, our study also reported higher odds of developing HPV infection among the women who used less than four sanitary pads in a day during their menstrual period than those who used more than four.

Many studies have reported staying in rural areas, poor socioeconomic status and illiteracy to be associated with HPV positivity [15, 45, 46]. Similarly, most of the HPV-positive participants in the research we conducted were from rural areas with most of them being unemployed and from the lower socioeconomic status. However, there was no statistically significant association found between these socio-demographic determinants and HPV infection in our study.

The HPV testing is found to be more sensitive than cytology (Pap) test alone [47, 48]. In the present study along with human papillomavirus (HPV) testing, cervical cytology (Pap test) was also done in combination as co testing. The clinical significance of co-testing in cervical cancer screening, is debatable. However co-testing could help to detect the precancerous changes of the cervix (eg, cervical dysplasia) associated with HPV and to initate the relevant treatment and further follow up [49].

HPV positivity and HR- HPV16 were found to be significantly associated with precancerous lesions like LSIL,HSIL and SCC as per the cytological reporting in the Pap smear in the current study which is in concordance with studies in Andhra Pradesh and Chennai, two other Indian states, found a comparable frequency of HR- HPV among cervical cancer patients [34, 35].

The limitation of the study was its cross-sectional nature. The behavioural and hygiene-related practices were self-reported by the study participants. Additionally, HPV retention or reinfection could not be elicited due to the need for extensive follow-up research. However, this is the first comprehensive study of its kind to provide the prevalence of HPV on this relatively unexplored island.

## Conclusion

Our study clearly showed that the women of these islands are at high risk of being infected with HPV, especially HR-HPV types 16. Precancerous lesions and squamous cell carcinoma of the cervix were found to be significantly associated with HPV positivity and high-risk HPV16. The outcome of the study emphasize stronger public health awareness programmes on cervical cancer and the need for introduction of HPV vaccine in these remote island.

## Data Availability

The data that support the findings of this study are not openly available due to reasons of sensitivity and are available from the corresponding author upon reasonable request.
